# Highly Efficient Multiscale Fog Collector Inspired by Sarracenia Trichome Hierarchical Structure

**DOI:** 10.1002/gch2.202100087

**Published:** 2021-09-12

**Authors:** Huawei Chen, Tong Ran, Kaiteng Zhang, Dengke Chen, Yang Gan, Zelinlan Wang, Lei Jiang

**Affiliations:** ^1^ School of Mechanical Engineering and Automation Beijing Advanced Innovation Center for Biomedical Engineering Beihang University Beijing 100191 China; ^2^ Laboratory of Bio‐inspired Smart Interface Science Technical Institute of Physics and Chemistry Chinese Academy of Sciences Beijing 100190 China

**Keywords:** bio‐inspired structures, fog harvesting, hierarchical microchannels, multiscale structures, Sarracenia trichome

## Abstract

Fog harvesting through bionic strategies to solve water shortage has drawn considerable attention. Recently, an ultrafast fog harvesting and transport mode was identified in Sarracenia trichome, which is mainly attributed to its superslippery capillary force induced by its unique hierarchical microchannel. However, the underlying effect of hierarchical microchannel‐induced ultrafast transport on fog harvesting and the multiscale structural coupling effect on highly efficient fog harvesting are still great challenges. Herein, a bionic Sarracenia trichome (BST) with an on‐demand regular hierarchical microchannel is designed using a one‐step thermoplastic stretching approach on a glass fiber bundle. The BST is engineered to harbor major channels confined by an inner gear pattern along with junior microchannels that are automatically assembled by the glass fiber monofilaments. The BST shows enhanced capillary condensation and fog harvesting performance, in part due to its coupling effect with a Janus membrane (JM). Hence, a highly efficient multiscale fog collector is developed, in which a gradient high‐pressure field is purposely formed to improve by threefold fog harvesting performance compared with a single‐scale structure. This easy manufacturing and low‐cost fog collector may represent a useful tool for harvesting fog water for production and living and pave the way for further investigations.

## Graphical abstract

A bionic Sarracenia trichome with an on‐demand regular hierarchical microchannel is designed to achieve excellent fog harvesting and transport properties as the real one. With the combination of Janus membrane, a highly efficient multiscale fog collector is developed, in which a gradient high‐pressure field is purposely formed to improve by threefold fog harvesting performance compared with a single‐scale structure.

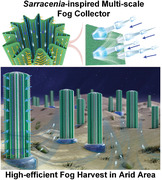

## 1 Introduction

As a promising way to solve urgent water shortage, harvesting water directly from fog has attracted worldwide attention in recent years.^[^ ^1^, ^2^, ^3^, ^4^, ^5^ ^]^ As an aerosol, the nano water droplets of fog normally tend to condense on the protruding solid surface, but the already condensed bulked water film will slow down further condensation.^[^ ^6^, ^7^, ^8^ ^]^ In nature, many biological samples have developed unique parts to solve the bulked water film‐restrained fog condensation,^[^ ^9^, ^10^, ^11^ ^]^ such as spider silk with spindle‐knots^[^ ^12^, ^13^, ^14^ ^]^ and cactus with cone spines.^[^ ^15^, ^16^, ^17^ ^]^ Their conical structures can directionally transport condensed water from the tip to the bottom, releasing the tip surface area for further fog condensation.^[^ ^18^, ^19^, ^20^, ^21^, ^22^ ^]^ Conical structures are usually combined with fog harps to construct fog collectors for highly efficient fog harvesting.^[^ ^23^, ^24^, ^25^, ^26^, ^27^, ^28^ ^]^ However, the velocity of directional water transport on these conical structures remains of ≈0.5 mm s^−1^, which limits further enhancement of fog harvesting by fog collectors.

**Figure 1:**
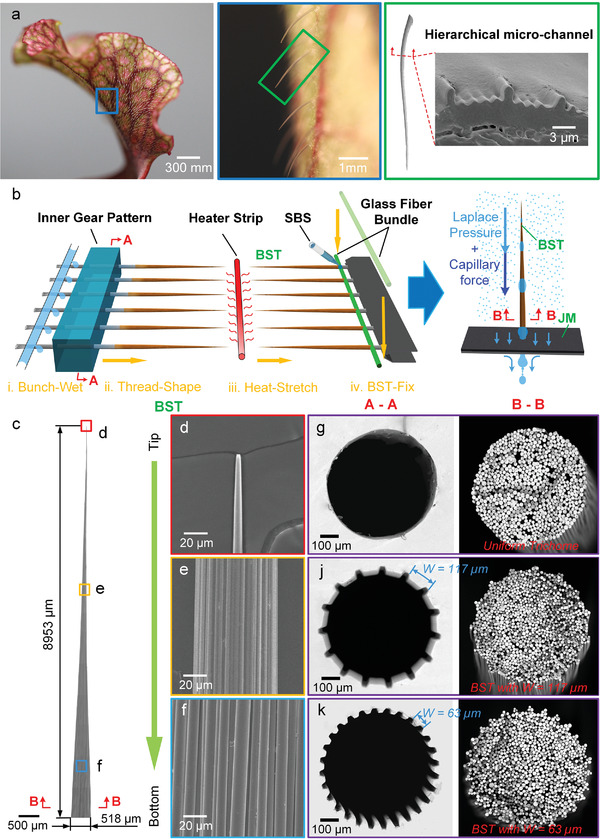
a) Optical images and SEM images of real Sarracenia trichome. b) One‐step thermo‐plastic stretching approach to fabricate BST, including four successive steps: i) bunch‐wet; ii) thread‐shape; iii) heat‐stretch; iv) BST‐fix. c) Overall view of BST. d–f) Partial enlarged details of BST. g–k) A–A is the cross‐section images of inner gear pattern, and B–B is the cross‐section images of BST. *W* stands for the spacing of inner gear pattern.

Fortunately, a more efficient fog harvesting and transport mode was discovered on Sarracenia trichomes that has a unique hierarchical microchannel structure around the needle‐shaped trichomes (**Figure** **1**a).^[^ ^29^ ^]^ A thin water film is automatically formed on the hierarchical microchannel structure to generate superslippery capillaries, which remarkably enhances the water transport capability and further reinforces the fog harvesting efficiency of trichomes. The hierarchical microchannel shows greater properties than the uniform microchannel, which can also aid on the development of new microfluidic systems.^[^ ^30^, ^31^, ^32^, ^33^ ^]^ However, the underlying dynamic mechanism of hierarchical microchannel‐induced ultrafast transport on fog harvesting is still ambiguous, and the multiscale structural coupling effect on fog harvesting performance is also a great challenge.

Herein, we propose an effective strategy to fabricate a bionic Sarracenia trichome (BST) using a one‐step thermoplastic stretching approach on a glass fiber bundle under the constraint of an inner gear pattern. The BST possesses an on‐demand hierarchical microchannel structure, whose major channels are confined by an inner gear pattern, as well as junior microchannels are automatically assembled by the glass fiber monofilaments. Its excellent gravity‐ignoring fog harvesting property was herein demonstrated, which was governed by a superslippery sliding mode, similar to the real Sarracenia trichome. The capillary condensation and fog harvesting theoretical model of BST was built to further discuss the dynamic impact of hierarchical microchannel‐induced ultrafast transport on fog harvesting. Moreover, the BST and Janus membrane (JM) coupling effects to enhance fog harvesting were discovered and discussed. Based on the coupling principle, a novel bio‐inspired multiscale fog collector was developed, which possessed a jungle structure formed by BST vertical on a JM plate. Wind blown into the fog collector would be trapped inside the jungle structure, which moisture would be harvested by the BST, thereby achieving highly efficient fog harvesting.

## 2 Results and Discussion

### 2.1 Fabrication and Characterization of the BST

Hierarchical microchannels with distributed high–low ribs played a pivotal role in the highly efficient fog harvesting system of Sarracenia trichomes (Figure 1a). A one‐step thermoplastic stretching approach was used to fabricate the BST, including four successive steps: bunch‐wet, thread‐shape, heat‐stretch, and BST‐fix (Figure 1b). Each bundle of glass fibers split from glass fiber rolls was first wetted with water to temporarily increase its surface viscosity. Next, the wetted glass fiber bundle was threaded through a hollow inner gear pattern to form a hierarchical microchannel structure on the surface. After that, the treated glass fiber bundle was heated to the plastic state and smoothly stretched to form BST, which had a homogeneous ratio of spacing and diameter in the cross‐section along the trichome. Finally, the BST was vertically adhered and bonded on a glass fiber bundle by styrenic block copolymers (SBS) to combine with the JM, which was a thin membrane that further enhanced the fog harvesting performance of BST by quickly transferring the harvested fog water from the superhydrophobic to the hydrophilic surface side.^[^ ^34^, ^35^, ^36^, ^37^ ^]^ To maintain strong adhesion of the glass fiber monofilaments, the heater strip was heated up to 800°C and installed at a 1 mm distance from the glass fiber bundle, and the stretching velocity was set to ≈10–14 mm s^−1^ (Figure S1, Supporting Information). The diameter of the glass fiber bundle split from the glass fiber roll was ≈520 µm slightly greater than the inner diameter of inner gear pattern (500 µm).

The BST overall possessed a perfect conical shape with a length of 8953 µm and a diameter of 518 µm at the bottom (Figure 1c). The partially enlarged observation of BST revealed that the microchannels were perfectly aligned from the tip to the bottom of the structure (Figure 1d–f). The cross‐section observation of the inner gear pattern (A–A) and BST (B–B) demonstrated that the gear shape of the inner gear pattern was perfectly transformed onto the surface of the BST (Figure 1g–k). The hierarchical microchannel on BST was one‐step formed by the integration of two types of microstructures, that is, large‐size gear shape of the inner gear pattern and small‐size glass fiber monofilaments. The former determined the major channels, whereas the small glass fiber monofilaments automatically assembled the junior channels. The high–low rib distribution of hierarchical microchannels could be adjusted by choosing an inner gear pattern with different spacing *W*. The BST with *W*  =  63 and 117 μm possessed 3–5 and 8–9 low ribs between neighboring high ribs, respectively. Therefore, this thermal‐plastic stretching approach enabled on‐demand fabrication of hierarchical microchannel structures on needle‐shaped trichomes.

### 2.2 Fog Harvesting and Transport Characterization of BST

**Figure 2:**
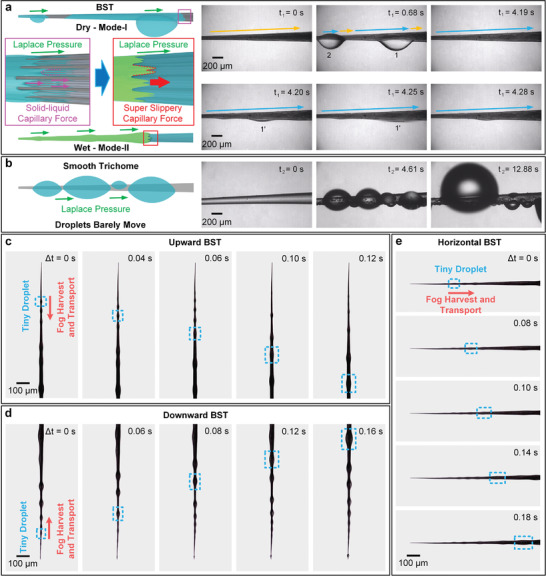
a) Fog harvesting and transport process of BST. The dry surface of BST is marked by yellow arrows, and the wet surface of BST is marked by blue arrows. b) Fog harvesting and transport process of smooth trichome without hierarchical microchannels. c–e) Fog harvesting and transport process of BST placed toward different directions. The direction of fog harvesting is marked by red arrows. The successive transported tiny droplets are marked by blue dotted frames.

The fog harvesting and transport properties of BST were assessed by placing BST inside a saturated fog flow with a velocity of ≈5 cm s^−1^ and humidity of over 90%. The BST with *W*  =  63 μm was used as the fog harvesting sample (**Figure** **2**a,c,e), while a smooth glass trichome fabricated by a capillary tube with the same conical shape was also prepared for comparison. These two types of trichomes were simultaneously covered with thin polyvinyl alcohol to obtain the same surface properties. The fog harvesting and transport on BST showed two distinct modes, similar to the real Sarracenia trichome. When the BST surface was dry (marked by yellow arrows), the fog condensed to form 1–2 droplets that were harvested and transported along the trichome in an intact whole water droplet. A thin water film was automatically formed on the hierarchical microchannels (marked by blue arrows) after these condensed water droplets slid through, as the cross‐section of BST A–A and B–B showed, which was Mode‐I (Figure 2a, (upward), Movie S1, Supporting Information). The capillary force induced by the hierarchical microchannel changed from a small solid–liquid capillary force to a large liquid–liquid super slippery capillary force. The fog harvesting and transport mode transformed from Mode‐I to Mode‐II, in which the subsequent transport of harvested fog water droplet 1′ was accelerated by superslippery sliding (Figure 2a, (downward), Movie S1, Supporting Information). The statistical velocity of fog harvesting and transport on wet BST was of ≈58.3 mm s^−1^, which is approximately hundred times faster than that of the cactus spine and spider silk. However, the smooth trichome without hierarchical microchannel completely lost its superior properties (Figure 2b; Movie S2, Supporting Information), indicating that these features were mainly dependent by the BST unique hierarchical microchannel. Similar to the real Sarracenia trichome, the BST also showed gravity‐ignoring fog harvesting and transport properties, and the ultrafast fog harvesting and transport phenomenon always occurred toward whichever direction the BSTs were placed (Figure 2c–e; Movie S3, Supporting Information). It should be noted that the inadequately stretched part of the glass fiber bundle tended to gather hysteretic droplets, which would remain on the trichome for a long time to hinder the BST continuous fog harvesting and transport. Therefore, the adhesion point of BST on JM was determined to be about ≈0.8 cm from the tip of BST to avoid the negative effect of inadequate stretched part, where JM could quickly transfer harvested fog water (Figure S2, Supporting Information).

### 2.3 Fog Harvesting Mechanism of the BST Hierarchical Microchannels

**Figure 3:**
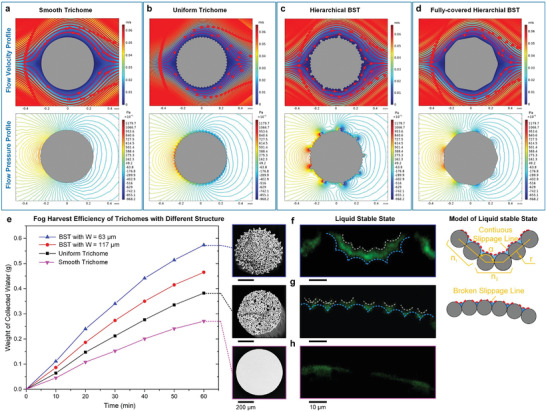
a–c) Flow velocity profile simulation and flow pressure profile simulation of trichomes with different structures. d) Flow velocity profile simulation and flow pressure profile simulation of fully‐covered hierarchical BST. e) Fog harvesting efficiency of trichomes with different structures. f‐h) The laser confocal transverse section images of liquid stable state on different trichomes are shown in the left part. The corresponding schematic models are respectively established in the right part. The extra concave curves of liquid film are marked by white dotted lines. The rough outlines of microchannels are marked by blue dotted lines. The slippage lines are marked by red dotted lines.

Fog harvesting of trichomes in fog flow generally consists of two steps: fog attraction and fog condensation.^[^ ^38^, ^39^, ^40^, ^41^ ^]^ Fog attraction determines the contact opportunities of tiny water droplets in air on the trichome surface, whereas fog condensation determines their capturing opportunities by the trichome surface. Thus, trichome structures should possess the capabilities of fog attraction and condensation as much as possible to enable higher fog harvesting efficiency. The fog flow around the trichome can be greatly influenced by its surface structure, in which the local pressure distribution around the trichome determines the fog attraction capability as a wind blow over.^[^ ^42^, ^43^, ^44^ ^]^ Generally, compared with the flat part, the hump part of the surface structure possessed higher local pressure to block wind flow, which would attract more fog onto the surface and provide more opportunities for fog harvesting. The flow velocity and pressure profiles around the different trichomes were simulated with a fog flow velocity of 5 cm s^−1^ (**Figure** **3**a–d). Compared with the smooth trichomes (Figure 3a), the microchannels on trichomes (Figure 3b,c) had a great impact on the fog flow due to its discontinuous surface structure, and the flow pressure was much more concentrated around the top of the ribs, indicating that the microchannels promoted the fog attraction of trichomes. In particular, the hierarchical microchannels of the hierarchical BST (Figure 3c) influenced the fog flow and the pressure concentration much more than the other two, and its maximum flow pressure became the largest among the three cases evaluated. Therefore, the hierarchical BST showed the best fog attraction properties.

The fog condensation phenomenon of the microchannel structure in a moist environment is called capillary condensation,^[^ ^45^, ^46^ ^]^ according to which a curved water surface always appears on the microchannel to dominate subsequent fog condensation. Because the vapor pressure above the curved water surface was lower than that above the flat‐water surface, it was easier to condense on the microchannel. Moreover, this fog condensation can be further promoted with increased surface curvature. Constructing and remaining the water surface with a larger curvature on the microchannel was important to improve fog condensation. The water film on a hierarchical microchannel usually had a larger curvature than that on a uniform microchannel, which made it more efficient for fog condensation. More importantly, the ultrafast superslippery sliding mode of the hierarchical microchannel could transport harvested fog water quickly, making the top of the ribs usually exposed in air and maintaining the optimal curvature of the water thin film. Assuming that the harvested fog water was not transported in time to fully cover the hierarchical microchannel, both the fog attraction capability and the fog condensation capability of the hierarchical BST should decline (Figure 3d), demonstrating that ultrafast water transport was very important for highly efficient fog harvesting. Therefore, the hierarchical microchannel could concurrently promote the capabilities of fog attraction and fog condensation on trichomes.

The fog harvesting efficiencies of different trichomes were herein tested (Figure 3e). The fog harvesting efficiency of BST with *W* = 63  μm was 111.8% and 50.1% higher than that of the smooth and uniform trichomes, respectively. The liquid stable states of these different trichomes were further observed by laser confocal microscopy (Figure 3f–h). Obviously, the liquid film upon hierarchical BST was thicker than that upon uniform trichome, which made the concave curves of the liquid film on hierarchical BST easier to form a continuous slippage line, thereby providing a multievel superslippery capillary force to enhance the water transport capability and enable large quantities of water transport (Figure 3f). However, with respect to the uniform trichome, the liquid film was much thinner and the slippage line appeared broken with a dispersive small water transport channel, which was easily fully buried in successive water transport (Figure 3g). Schematic models of these liquid stable states could be derived (Figure 3f,g). The high–low rib distribution of hierarchical microchannels on the BST may significantly influence its water transport capability and its fog harvesting efficiency. Although the BST with *W*  =  117 μm had higher fog harvesting efficiency than smooth trichomes and uniform trichomes, its fog harvesting efficiency was 18.9% lower than that of BST with *W*  =  63 μm. The water transport capability of wetted hierarchical microchannels can be represented by its flux *Q* ^(*s*)^, which was derived as follows^[^ ^29^ ^]^ (further analysis in Figure S3 and Equations (S1)–(S6), Supporting Information)

(1) $$\begin{array}{c} \begin{array}{c} \;Q^{\left(s\right)}=\frac{1}{2}\;D^{\left(s\right)}\bar{S}\;\sqrt{\frac{r\gamma }{\eta t}},\\ D^{\left(s\right)}=\sqrt{\frac{4\cdot \left(2n_{1}+n_{2}-2+\cos\theta \right)\cdot \pi +16n_{1}\cdot \cos\alpha -8n_{2}+8}{\left[8n_{1}\cdot \sin\alpha \cdot \left(n_{2}-n_{1}\cdot \cos\alpha -1\right)+4n_{2}-8n_{1}\cdot \cos\alpha -4-\left(n_{2}+2n_{1}-1\right)\cdot \pi \right]\beta^{\left(s\right)}}} \end{array} \end{array}$$

where *D* ^(*s*)^ is the geometrical coefficient of the major microchannel, $\bar{S}$ is the summation of the cross‐sectional area of the total flux, *r* is the radius of a single glass fiber monofilament, γ is the interfacial tension, η is the dynamic viscosity of the fluid, θ is the Young equilibrium contact angle, β^(*s*)^ is the dimensionless flow resistance under the flow boundary condition, *n*_1_ and *n*_2_ are the number of glass fiber monofilaments in the side wall and bottom of the hierarchical microchannel, respectively, and α is the included angle between the side wall and bottom of the hierarchical microchannel which generally ranges from 90° to 180°.

By theoretical analysis, the best water transport property existed on the hierarchical microchannel with α  =  90°, whereas this water transport property generally decreased as α gradually approached 180° (Figure S3a, Supporting Information). The increase in *n*_1_ was beneficial for enhancing the water transport capability of the hierarchical microchannel, and the hierarchical microchannel with *n*_2_ ranging from 3 to 4 exhibited the best water transport performance (Figure S3b–d, Supporting Information). BST with *W*  =  63 and 117  μm possessed the same α and *n*_1_, whereas *n*_2_ of these two cases was different, ranging from 3 to 5 and from 8 to 9, respectively. Therefore, BST with *W*  =  63  μm was more capable of water transport and fog harvesting. The design regulation of the hierarchical microchannel structure on BST was also suitable for trichomes fabricated using other tiny fibers.

### 2.4 Fog Harvesting Promotion of BST

**Figure 4:**
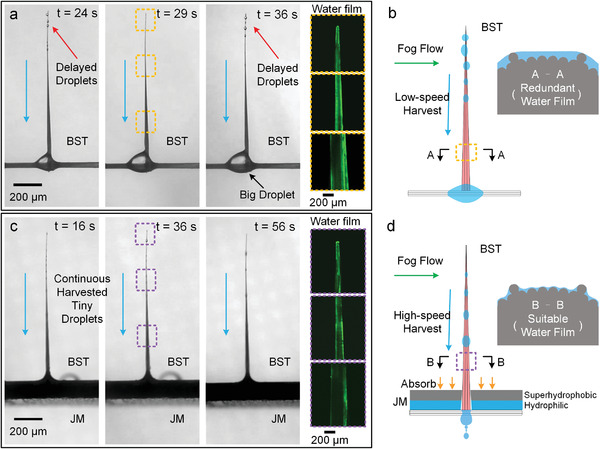
a) Fog harvesting process of BST vertically boned onto glass fiber bundle. The water film of BST from the tip to the bottom are observed by plane laser confocal photograph which are marked by yellow dotted lines. b) Schematic model of fog harvesting process of BST without JM. A–A shows the cross‐section of BST without JM. c) Fog harvesting process of BST+JM. The water film states of BST+JM from top to bottom are observed by plane laser confocal photograph which are marked by purple dotted lines. d) Schematic model of fog harvesting process of BST with JM. B–B shows the cross‐section of BST with JM.

The characteristic structure of hierarchical microchannels was better to be wetted by a uniform liquid thin film in the duration of highly efficient fog harvesting. As for the BST vertically bonded onto the glass fiber bundle, the harvested fog water gradually gathered around the junction point to form a large hysteretic droplet, which consequently resulted in water film thickening upon hierarchical microchannels. The thickened water film should recede the water transport capability of the hierarchical microchannel on BST (Figure S4, Supporting Information), which gave rise to the appearance of several delayed droplets near the tip (**Figure** **4**a; Movie S4, Supporting Information). JM provided a possible strategy to quickly transfer hysteretic harvested fog water on the BST. Regularly arranged through‐holes with a radius of ≈1 mm were drilled on the JM to directly combine with the BST. JM and BST formed a coupling multiscale fog harvesting system, which greatly improved the fog harvesting efficiency of BST. With the assistance of the JM, the fog water harvested by the BST was quickly transported from the tip to the bottom without the appearance of a large hysteretic droplet around the junction point (Figure 4c; Movie S3, Supporting Information). The water thin film upon BST (Figure 4a, yellow dotted lines) and BST+JM (Figure 4c, purple dotted lines) were observed by fluorescence microscopy. The light intensity of fluorescence clearly demonstrated that the water film upon BST was thicker than that upon BST+JM. Thus, JM effectively transferred the harvested fog water and decreased the redundant water film upon the hierarchical microchannels to promote the water transport capability and fog harvesting efficiency of the BST (Figure 4b,d).

### 2.5 Construction of a Fog Collector Based on BST and JM

**Figure 5:**
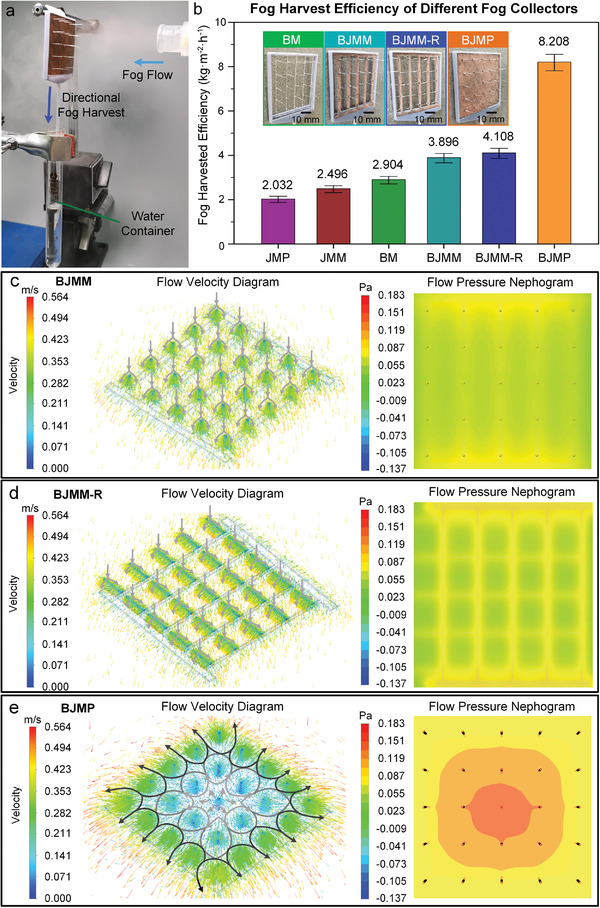
a) Fog collectors based on BST and JM are experimented in saturated fog flow, the harvested fog water is directional collected into the water container. b) Six types of fog collectors are respectively experimented, i.e., JM plate (JMP), JM mesh (JMM), BST mesh (BM), vertical BST+JM mesh (BJMM‐V), horizontal BST+JM mesh (BJMM‐H), and BST+JM plate (BJMP). Flow velocity diagram and flow pressure nephogram of BJMM‐V c), BJMM‐H d) and BJMP e) is proposed with the flowing wind velocity of 0.5 m s^−1^. The flow velocity diagram shows the direction of wind, which is marked by grey arrows.

To investigate the effects of BST and JM coupling on fog harvesting performance, different types of fog collectors based on BST and JM were constructed and experimented in a saturated fog flow of ≈0.5 m s^−1^ and humidity of over 90%, with the harvested fog water being collected into the water container beneath the fog collectors (**Figure** **5**a). Six different types of fog collectors were prepared: JM plate (JMP), JM mesh (JMM), BST mesh (BM), vertical BST+JM mesh (BJMM‐V), horizontal BST+JM mesh (BJMM‐H), and BST+JM plate (BJMP). All JMs had the same microstructure regardless of the plate or mesh. BJMM‐V and BJMP were fabricated by pushing BST through the drilled through‐holes, and BJMM‐H was obtained by twirling the JM mesh and BST of BJMM‐V from vertical to horizontal (Figure S5, Supporting Information). Generally, JMM showed a 22% higher fog harvesting efficiency than JMP. The combination of BST with JM commonly promoted the fog harvesting efficiency; for example, the efficiency of BJMM‐V was improved by 56.1% compared with that of JMP. The change in the BST orientation on the JM hardly affected the fog harvesting efficiency, with only a 5.4% difference. However, the utilization of the JM plate greatly enhanced the overall fog harvesting efficiency, with BJMP achieving 110.7% and 99.8% higher fog harvesting efficiency than BJMM‐V and BJMM‐H, respectively (Figure 5b). Compared with the single‐scale structure, that is, JMP, JMM, and BMM, BJMP showed a threefold higher fog harvesting efficiency. The BST and JM coupling effects were further investigated by fluid simulation at a wind velocity of 0.5 m s^−1^, in which the flow velocity and flow pressure in the BJMM‐V (Figure 5c), BJMM‐H (Figure 5d), and BJMP (Figure 5e) were explored. The wind blew towards the axial direction of the BST on BJMM‐V, whereas it blew towards the radial direction of the BST on BJMM‐H. The BST on BJMM‐H had a much greater exposure area to the wind than that on BJMM‐V, resulting in higher fog harvesting efficiency. For BJMP, the wind direction was greatly changed from axial to radial, and the wind was twisted to blow through the jungle structure of the BST on the JM plate. Thus, the JM plate reinforced the interaction between BST and flowing wind to greatly enhance the contact opportunity of moisture on BST, that is, to fully mobilize the fog harvesting property of the BST. The flow pressure nephogram further demonstrated that the pressure distributions on the BJMM‐V and BJMM‐H were uniform and similar to the low‐pressure field, but that on BJMP completely changed with the gradient high‐pressure field. The pressure distribution on the BJMP radiated from the center to the edge, and the maximum pressure of the BJMP was ≈0.183 Pa higher than the 0.1 Pa detected in the BJMM‐V and BJMM‐H. Therefore, the fog should be much more easily captured by BJMP, as the combination of the BST and the JM plate was more beneficial for fog harvesting.

BJMM‐V and BJMM‐H adopted a widely used fog collection system, which combined BST with JM‐fabricated fog harps.^[^ ^27^, ^28^ ^]^ For this type of fog collection system, the wind quickly flowed through the intervals between neighboring harps, in which the wind only blew through a single BST. For BJMP, the wind was twisted inside the jungle structure of the BST to contact with several BSTs, and the fog harvesting was enormously enhanced. This new type of collection system paves the way for new potential ideas of highly efficient fog harvesting.

**Figure 6:**
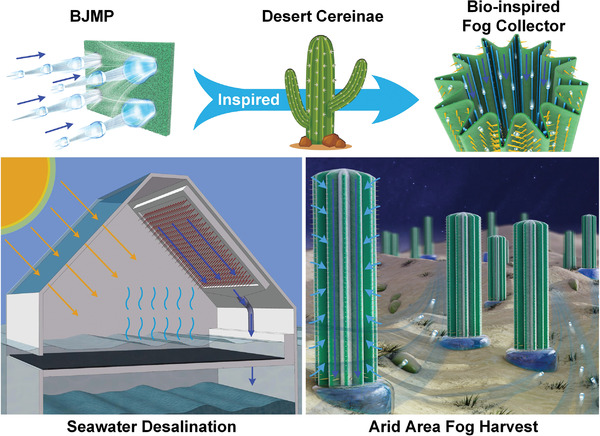
The large‐area flat BJMP can be used in seawater desalination. Inspired by desert cereinae, bio‐inspired fog collector can be constructed to harvest fog water in arid area.

Based on BJMP, a novel concept of highly efficient multiscale fog collector could be proposed for different harvesting requirements (**Figure** **6**). For seawater desalination, the large‐scale flat BJMP could be hung up on the ceiling of a semi‐closed space that only had a water inlet under the sea surface. The BJMP could continuously harvest moisture that was evaporated from seawater by sunlight and transport it to the container. Moreover, under the inspiration of desert cereinae, bio‐inspired fog collectors could also be built for highly efficient fog harvesting in arid areas.

## 3 Conclusion

In summary, a BST with on‐demand regular hierarchical microchannels was successfully fabricated using a one‐step thermo‐plastic stretching approach on a glass fiber bundle, in which the major channels were confined by an inner gear pattern and the junior microchannels were automatically assembled by a glass fiber monofilament. The BST showed ultrafast gravity‐ignoring fog harvesting and transport properties in the manner of superslippery sliding, similar to the real Sarracenia trichome. In addition, the dynamic influencing mechanism of hierarchical microchannel‐induced ultrafast transport on fog harvesting was explored via capillary condensation theory and a theoretical fog harvesting model of BST. The design regulation of hierarchical microchannels on trichomes fabricated using tiny fibers has also been proposed. The BST and JM coupling effect was discovered, and the coupling principle was proposed to enhance the fog harvesting performance. Finally, a new, highly efficient multiscale fog collector was constructed based on the combination of BST and JM plates, in which a gradient high‐pressure field was purposely formed to improve by threefold fog harvesting performance compared with that of a single‐scale structure. This low‐cost fog collector was easy to manufacture, representing a basis for enhanced fog water harvesting systems in diverse areas, such as seawater desalination and arid areas.

## 4 Experimental Section

### Preparation of Materials

The glass fiber bundle was purchased from Taishan Fiberglass Inc. (Taian, China). Polydimethylsiloxane (PDMS) was purchased from Dow Silicones Co., Ltd. (Zhangjiagang, China). The copper film was purchased from Jiashide Inc. (Suzhou, China).

### Fabrication of BST

The heater strip was purchased from Ruiqi Technic Corporation (Jiangsu, China). The material of the heater strip was Cr20Ni80 (2080) with a diameter of 1 mm. The working voltage and power of the heater strip were 220 V and 2000 W, respectively. The inner gear patterns were duplicated using a PDMS prepolymer in the template of the columns with a specific gear shape. The column was fabricated by 3D printing (nanoArch P140, BMF Material Technology Inc., Shenzhen, China) with an inner diameter of 500 µm. BST and glass fiber bundles were fixed using 0.8 µL SBS solution, which was dissolved into a 10 wt% solution with methylbenzene.

### Microstructure Observation of BST

The BST was first fixed on the sample plate in the axial direction, then was observed step‐by‐step from the tip to the bottom by scanning electron microscopy (JSM‐6010, JEOL, Tokyo, Japan) with a 10.0 KV accelerating voltage. To observe the cross‐section of the BST, the BST was first immersed in liquid nitrogen for over 1 min to increase its fragility; then, the BST was quickly snapped near the bottom using a sharp razor. Finally, the broken BST was fixed on the sample plate in the vertical direction. Cross‐sectional images of BST were collected by scanning electron microscopy (JSM‐6010) with a 10.0 KV accelerating voltage.

### Fabrication of the JM

The JM was fabricated based on copper foam (Longshengbao Inc., Kunshan, China) with a thickness of 1 mm. The copper foam was first cut to the desired shape and drilled regularly arranged through‐holes with a radius of ≈1 mm and with 1 cm intervals. The copper foam was then immersed in a silver nitrate solution (1 wt%) for 10 s to increase its hydrophilic property, and then silica superhydrophobic coating was sprayed on one side of the copper foam.

### Observation of Fog Harvesting and Transport Process

The fog harvesting and transport processes of BST and smooth trichome in Figure 2a,b were investigated by keeping the trichome inside a saturated fog flow with a velocity of ≈5 cm s^−1^ and humidity over 90% (by an ultrasonic humidifier using Milli‐Q water) under a microscope (BX51; Olympus, Tokyo, Japan) coupled with a high‐speed camera collecting images at 200 fps (I‐speed LT, Olympus). Polyvinyl alcohol was dissolved in hot water at 90 °C and then the liquid supernatant was removed to decorate the BST and the smooth trichome. The fog harvesting and transport process of trichomes in Figures 2c–e and 4a,c were investigated by keeping the trichomes inside a saturated fog flow with a velocity of ≈5 cm s^−1^ and humidity over 90% (by an ultrasonic humidifier using Milli‐Q water) under a microlens via a high‐speed camera collecting images at 50 fps (I‐speed LT, Olympus).

### Fog Harvesting Experiments of Different Trichomes

The fog harvesting efficiency of different trichomes in Figure 3e was investigated by keeping the trichomes inside a saturated fog flow with a velocity of ≈5 cm s^−1^ and humidity over 90% (using an ultrasonic humidifier with Milli‐Q water). A water container was deposited to collect the harvested fog water. The weight of the harvested fog water was measured using an analytical balance (BSM‐220.4, Zhuojing, Shanghai) per 10 min.

### Laser Confocal Observation

A laser confocal microscope (LSM 780; Zeiss, Oberkochen, Germany) was used to observe the liquid stable state on different trichomes (Figure 3f–h). The liquid stable state could only be observed by the 3D imaging function of the laser confocal microscope, which generally took several minutes to record, and it was difficult to maintain a stable water thin film due to water evaporation. The PDMS prepolymer, in which the fluorescence labels were uniformly dispersed, was used to imitate the water. The fluorescence label used was fluorescein sodium. Owing to the low liquidity of PDMS, the waiting time after pouring the PDMS on the tip of the trichomes was extended to ≈2 h. Liquid stable states were observed at the bottom of the trichomes. As for the laser confocal observation of water film on BST and BST+JM in Figure 4a,c, only one image that crossed the central axis of the BST was necessary. The time taken to obtain a single plane laser confocal image was less than 1 s, and water evaporation could be ignored. The BST and BST+JM were first deposited inside a saturated fog flow with a velocity of ≈5 cm s^−1^ and humidity over 90% to harvest fog water for a period of time (by an ultrasonic humidifier using 1 wt% fluorescein sodium mixed Milli‐Q water). The wetted BST and BST+JM were quickly deposited under a laser confocal microscope (LSM 780) to observe their water film.

### Experiments of Fog Collectors

The fog collectors in Figure 5b were investigated in a saturated fog flow with a velocity of ≈0.5 m s^−1^ and humidity over 90% (by a high‐power ultrasonic humidifier using Milli‐Q water). The outline frame to hold the fog harvesting structure was manufactured by 3D printing that was hollow inside (nanoArch P140, BMF Material Technology Inc., Shenzhen, China). The harvested fog water was gathered by the hollow frame and transported to the water container through the water pipe.

## Conflict of Interest

The authors declare no conflict of interest.

## Supporting information

Supporting Information

Supplemental Movie 1

Supplemental Movie 2

Supplemental Movie 3

Supplemental Movie 4

## Data Availability

The data that supports the findings of this study are available in the Supporting Information of this article.
