# Supplementary material for: Highly Efficient Multiscale Fog Collector Inspired by Sarracenia Trichome Hierarchical Structure
Source: Glob Chall. 2021 Sep 12;5(12):2100087. doi: 10.1002/gch2.202100087 (PMC8671618; doi:10.1002/gch2.202100087)
Supplement: Supplementary file 1 — Supporting Information [file GCH2-5-2100087-s001.pdf]

© 2021 Wiley-VCH GmbH

Global Challenges
Open Access

Supporting Information

for *Global Challenges,* DOI: 10.1002/gch2.202100087

Highly Efficient Multiscale Fog Collector Inspired by Sarracenia Trichome Hierarchical Structure

Huawei Chen, Tong Ran, Kaiteng Zhang, Dengke Chen, Yang Gan, Zelinlan Wang, and Lei Jiang*

Supplementary Information For

High-efficient Multi-scale Fog Collector Inspired on *Sarracenia* Trichome Hierarchical-structure

This file includes:

Supplementary text

Supplementary Figure S1 to Supplementary Figure S5

Supplementary Table I

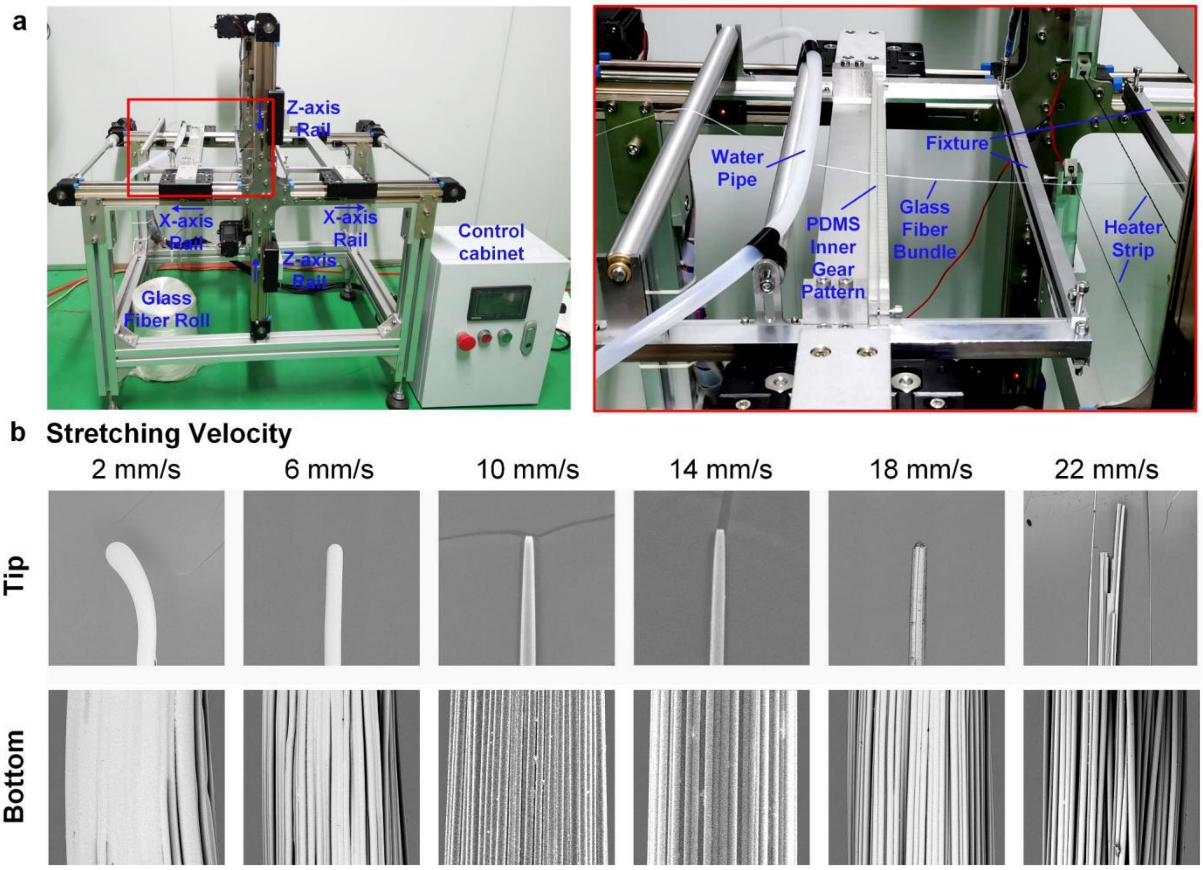

Figure S1. Exploration of BST fabrication. (a) The BST fabricating device is manufactured based on four linear guide modules which has two X-axis rails and two Z-axis rails. The X-axis rails are used to stretch the glass fiber bundle, and the Z-axis rails are used to control the distance between heater strip and glass fiber bundle. The movements of the linear guide modules are controlled by control cabinet. In successive fabrication of BST, the glass fiber bundle is constantly split from the the glass fiber roll. The split glass fiber bundle is wetted by water pipe with little holes around. The PDMS inner gear pattern is used to confine the hierarchical micro-channels around the glass fiber bundle. At last, the confined glass fiber bundle is heated and stretched. (b) To keep strong adhesion of glass fiber monofilaments without breaking the micro-channel morphology of glass fiber bundle, the heating temperature and stretching velocity was deliberately determined. Under the condition that the heater strip heated up to 800℃ is installed at a distance of 1 mm from glass fiber bundle, the influence of stretching velocity on BST is explored. When the stretching velocity is low, the tip of BST bends and losses the conical shape, meanwhile, the glass fiber melts too much that the BST losses micro-channel morphology at the bottom (the stretching

velocity is 2 mm/s and 6 mm/s). When the stretching velocity is too high, the glass fiber can not fully melt and firmly adhere with others that the fracture of glass fiber occurs at tip and the separation of BST occurs at bottom (the stretching velocity is 18 mm/s and 22 mm/s). When the stretching velocity is 10 mm/s and 14 mm/s, the BST possesses proper structure that the tip of BST presents superior conical shape and the bottom of BST gets preferable micro-channel morphology. Therefore, the stretching velocity ranging from 10 mm/s to 14 mm/s is finally determined to fabricate BST.

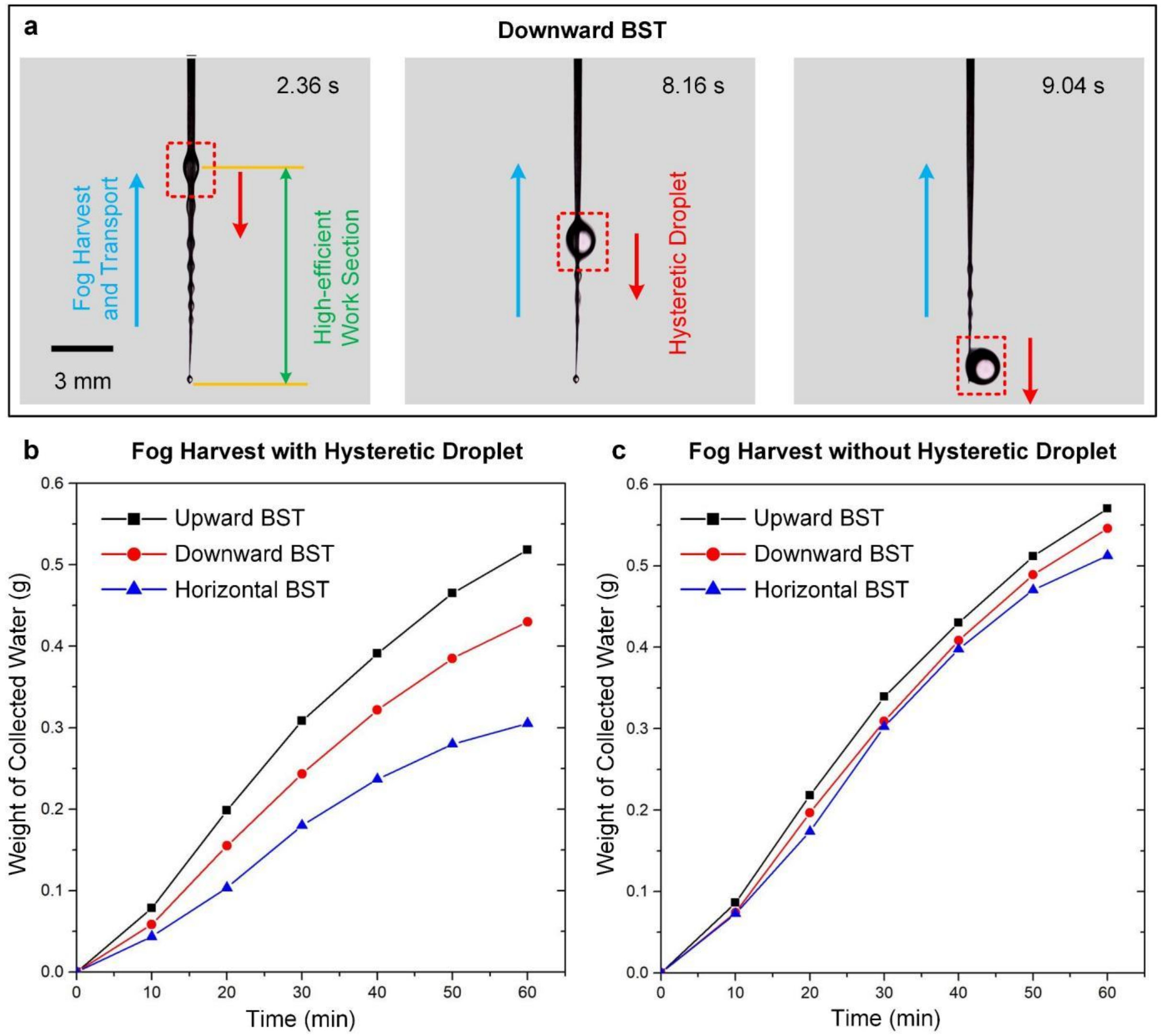

Figure S2. Fog harvesting and transport of BST placed toward different directions. (a) Fog harvesting and transport of downward BST. Under the circumstances that the harvested fog water is not transferred in time, a big hysteretic droplet gathered at the position with small conical angle variation of BST. With the hysteretic droplet growing, the gravity little by little takes charge, that the hysteretic droplet moves toward the direction of gravity. Before the gravity takes charges of hysteretic droplet, the ultrafast gravity-ignoring fog harvesting part on BST is defined as high-efficient work section, which is the length between the tip of BST and the initial gathering position of hysteretic droplet on BST (marked by yellow and green lines). By statistics, the high-efficient work section of downward BST is about 0.8 cm which determines the adhesion point of BST on JM. (b) Without the hysteretic droplet transferred, the fog harvesting efficiency of BST placed toward different directions shows huge differences that the upward BST possesses the best fog harvesting performance and the

horizontal BST possesses the worst. (c) With the hysteretic droplet transferred by JM, the fog harvesting efficiency gaps of BST placed toward different directions greatly reduce. It is noted that the fog harvesting efficiency of upward BST also promotes with hysteretic droplet transferred. It can be concluded that the rapid transferring of harvested fog water is beneficial to promote the fog harvesting efficiency of BST.

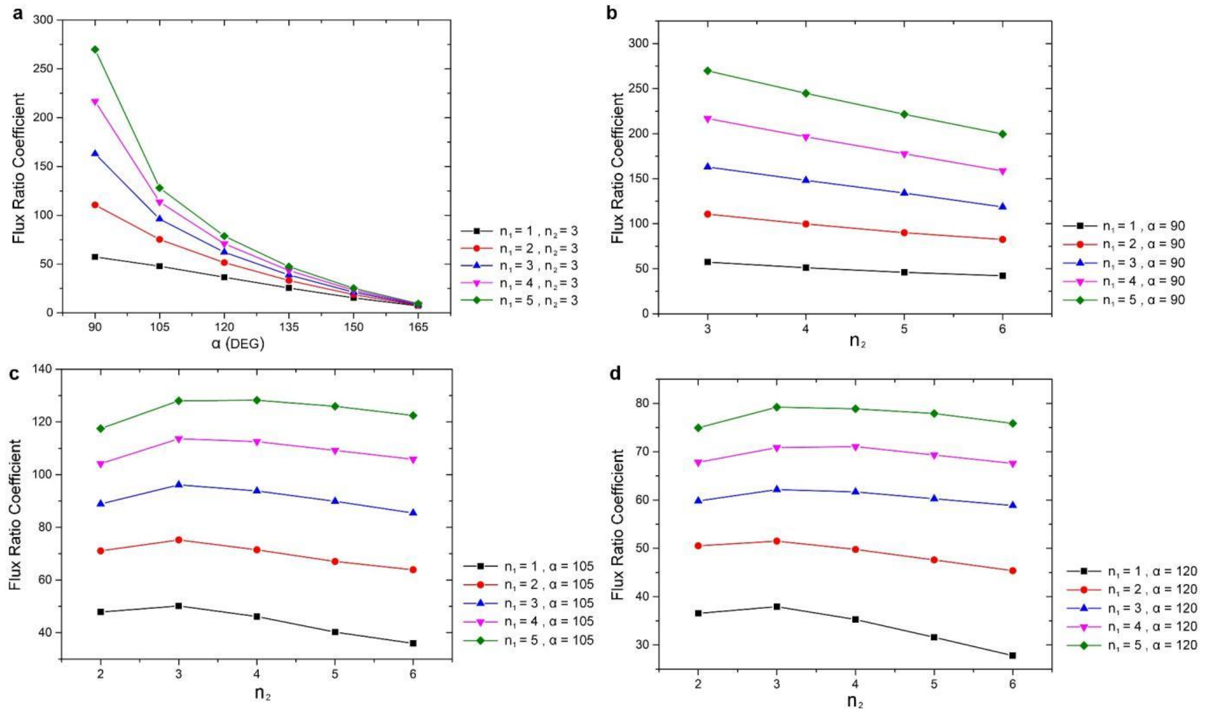

Figure S3. Analysis and calculation of water transport property of hierarchical micro-channel with different structure. (a) Flux in hierarchical micro-channel with $n_2 = 3$ and different inclined angle α, n_1. (b) Flux in hierarchical micro-channel with inclined angle $\alpha = 90°$ and different n_1, n_2. (c) Flux in hierarchical micro-channel with inclined angle $\alpha = 105°$ and different n_1, n_2 (d) Flux in hierarchical micro-channel with inclined angle $\alpha = 120°$ and different n_1, n_2.

The free energy of hierarchical micro-channel formed by glass fiber is written as

$$A = d^{(s)} \cdot r \cdot [-(2n_1 + n_2 - 2 + cos\theta) \cdot \pi - 4n_1 \cdot cos\alpha + 2n_2 - 2] \tag{S1}$$

The dissipation can be written as

$$\Phi = \frac{1}{2}\frac{\eta}{r^2}\beta^{(s)} S d^{(s)} \dot{d^{(s)}} \tag{S2}$$

where

$$S = 4n_1 \cdot sin\alpha \cdot (n_2 - n_1 \cdot cos\alpha - 1) + 2n_2 - 4n_1 \cdot cos\alpha - 2 - \frac{1}{2} \cdot (n_2 + 2n_1 - 1) \cdot \pi r^2 \tag{S3}$$

The evolution equation is given by

$$\frac{\delta \mathcal{R}}{\delta \dot{d}_3} = r \cdot [-2n_1 \cdot (cos\alpha + 1) - \pi + 2\alpha + 3cot\frac{\alpha}{2} - \pi \cdot cos\theta] + \frac{1}{2}\frac{\eta}{r^2}\beta^{(s)} S d^{(s)} \dot{d^{(s)}} = 0 \tag{S4}$$

The solution is

$$d^{(s)} = D^{(s)}\sqrt{\frac{r\gamma}{\eta}t},$$

$$D^{(s)} = \sqrt{\frac{4\cdot(2n_1+n_2-2+cos\theta)\cdot\pi+16n_1\cdot cos\alpha-8n_2+8}{[8n_1\cdot sin\alpha\cdot(n_2-n_1\cdot cos\alpha-1)+4n_2-8n_1\cdot cos\alpha-4-(n_2+2n_1-1)\cdot\pi]\beta^{(s)}}} \tag{S5}$$

The water transport is mainly attributed by top major filling and the flux can be given by

$$Q^{(s)} = \dot{d}^{(s)}\bar{S} = \frac{1}{2}D^{(s)}\bar{S}\sqrt{\frac{r\gamma}{\eta t}} \tag{S6}$$

where $\bar{S}$ is summation of the cross-section area of total flux. We use $D^{(s)}\bar{S}$ as flux ratio coefficient to compare the flux of hierarchical micro-channel with different structure. By theoretical analysis, the best water transport property existed on the hierarchical micro-channel with $\alpha = 90°$, while this water transport property generally decreased with α gradually approaching $180°$ (Figure S3a). It can also be concluded that the increase of n_1 is in favor of water transport enhancement (Figure S3a), and the hierarchical micro-channel with n_2 ranging from 3 to 4 possesses the best performance of water transport (Figure S3b-d).

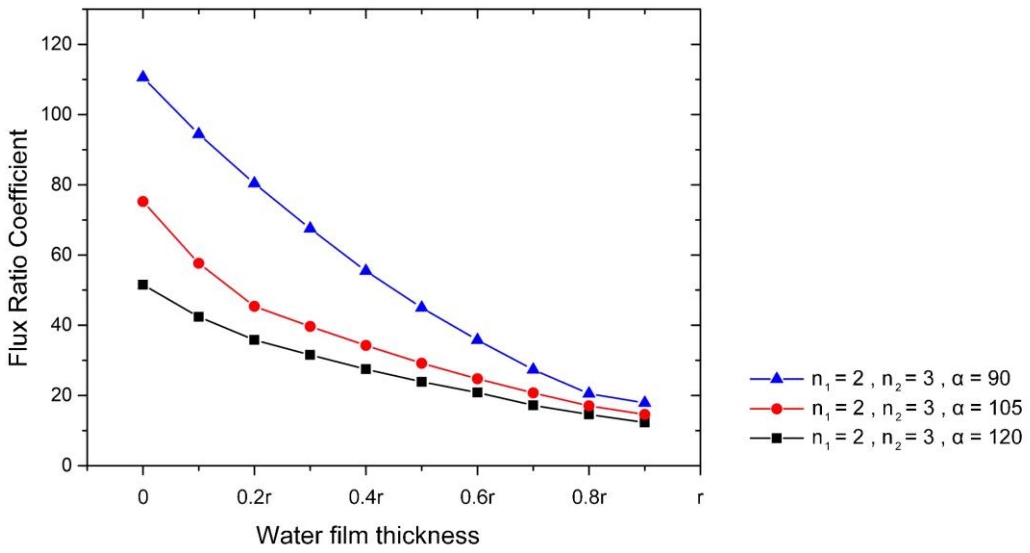

Figure S4. Flux in hierarchical micro-channel influenced by water film thickness. The thickness of water film corresponds to the boundary layer in water transport. The increase of boundary layer leads to the decrease of $\bar{S}$ and $\beta^{(s)}$ in Eq. S5-6. Three types of hierarchical micro-channel with the same n_1 and n_2 but different inclined angle α are used to analyze the influence of water film thickness on flux. By simulation, the numerical results of $\bar{S}$ and $\beta^{(s)}$ according to different water film thickness $\delta^{(s)}$ is obtained, which is shown in Table I. With the increase of water film thickness, the flux ratio coefficient gradually decreases, which means the decline of flux in hierarchical micro-channel.

Table I

$\delta^{(s)}$	$\alpha = 90$			$\alpha = 105$			$\alpha = 120$		
	$\bar{S}$	$\beta^{(s)}$	$D^{(s)}\bar{S}$	$\bar{S}$	$\beta^{(s)}$	$D^{(s)}\bar{S}$	$\bar{S}$	$\beta^{(s)}$	$D^{(s)}\bar{S}$
0	259.092	5.395	110.573	249.919	4.847	75.2297	237.157	4.721	51.510
0.1r	221.181	5.287	94.394	219.439	4.750	51.6463	218.201	4.626	47.3929
0.2r	201.799	5.179	80.3936	188.375	4.653	45.389	156.822	4.532	43.8303
0.3r	186.717	5.071	67.5202	158.211	4.557	40.6371	136.947	4.437	38.5545
0.4r	142.591	4.963	55.4635	129.960	4.460	37.2497	118.333	4.343	34.486
0.5r	129.243	4.855	44.9709	105.374	4.363	34.1805	100.819	4.248	29.5874
0.6r	106.565	4.747	35.7802	85.512	4.266	31.7501	83.839	4.154	24.8336
0.7r	94.489	4.639	29.349	71.680	4.169	27.7467	64.0846	4.060	20.2107
0.8r	87.167	4.532	27.530	58.915	4.072	19.052	45.7635	3.965	17.620
0.9r	61.966	4.424	24.890	47.042	3.975	17.615	41.9195	3.871	13.318

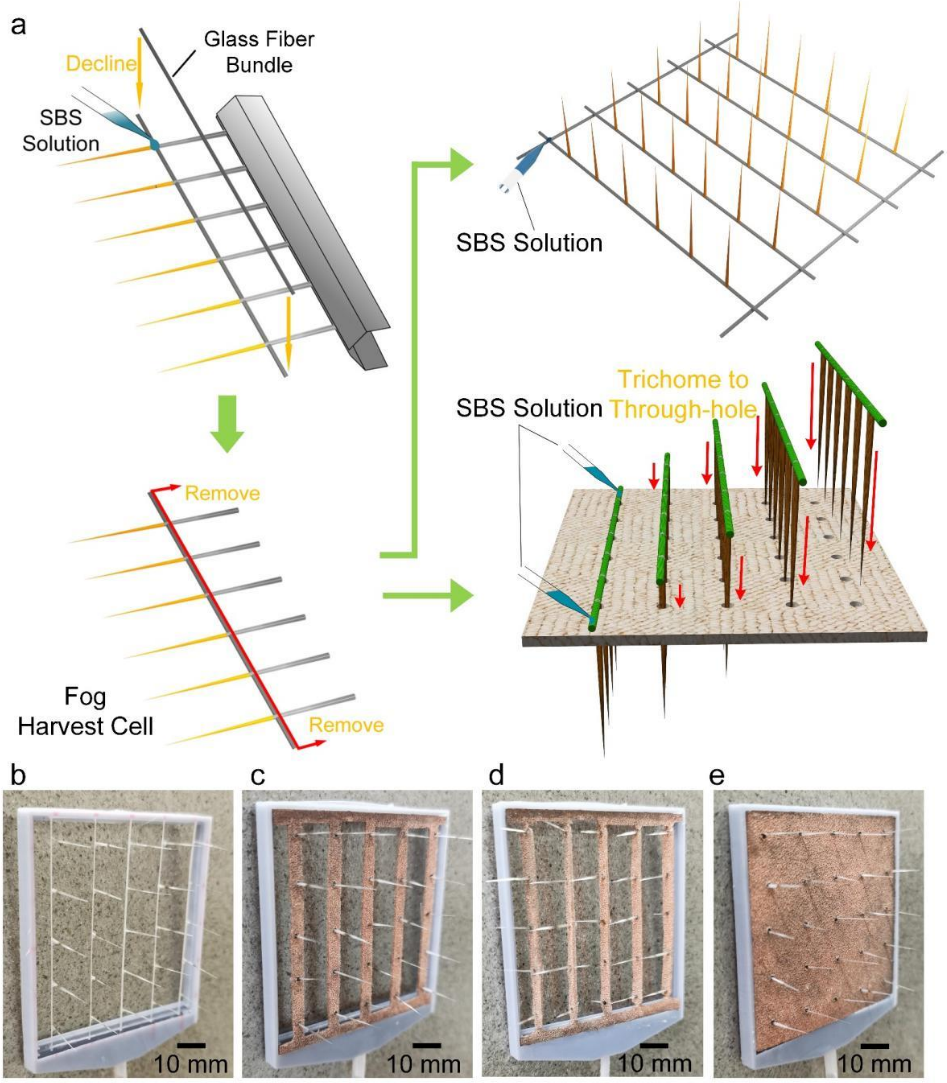

Figure S5. Fabrication of BST fog harvesting mesh. (a) By use of SBS, the BST is vertically boned upon glass fiber bundle. The interval between two neighboring BST is ~1 cm, and the fixed position of glass fiber bundle is 0.8 cm from tip of BST which is determined by the high-efficient work section. After the BST and the glass fiber bundle are boned together, the redundant part of BST is cut off, achieving fog harvesting cell. The fog harvesting cells are paralleled boned upon other two glass fiber bundles with the interval of 1 cm, achieving BST mesh (BMM, b). By quick matching the trichomes with the through-holes, vertical BST+JM mesh (BJMM-V, c) and BST+JM plate (BJMP, e) is manufactured. Both the ends of fog harvesting cells are fixed on JM by SBS. By twirled the JM mesh and BST of BJMM-V from vertical to horizontal, horizental BST+JM mesh was manufactured (BJMM-H, d).
